# Comparison of Respiratory Disease Prevalence among Voluntary Monitoring Systems for Pig Health and Welfare in the UK

**DOI:** 10.1371/journal.pone.0128137

**Published:** 2015-05-28

**Authors:** J. I. Eze, C. Correia-Gomes, J. Borobia-Belsué, A. W. Tucker, D. Sparrow, D. W. Strachan, G. J. Gunn

**Affiliations:** 1 Scottish Rural College, Kings Building, West Mains Road, Edinburgh, United Kingdom; 2 MossVet, 34 Seagoe Industrial Estate, Portadown, Craigavon, County Armagh, Northern Ireland, United Kingdom; 3 Department of Veterinary Medicine, University of Cambridge, Cambridge, United Kingdom; 4 Boehringer Ingelheim Vetmedica, Bracknell, United Kingdom; University of Bari, ITALY

## Abstract

Surveillance of animal diseases provides information essential for the protection of animal health and ultimately public health. The voluntary pig health schemes, implemented in the United Kingdom, are integrated systems which capture information on different macroscopic disease conditions detected in slaughtered pigs. Many of these conditions have been associated with a reduction in performance traits and consequent increases in production costs. The schemes are the Wholesome Pigs Scotland in Scotland, the BPEX Pig Health Scheme in England and Wales and the Pig Regen Ltd. health and welfare checks done in Northern Ireland. This report set out to compare the prevalence of four respiratory conditions (enzootic pneumonia-like lesions, pleurisy, pleuropneumonia lesions and abscesses in the lung) assessed by these three Pig Health Schemes. The seasonal variations and year trends associated with the conditions in each scheme are presented. The paper also highlights the differences in prevalence for each condition across these schemes and areas where further research is needed. A general increase in the prevalence of enzootic pneumonia like lesions was observed in Scotland, England and Wales since 2009, while a general decrease was observed in Northern Ireland over the years of the scheme. Pleurisy prevalence has increased since 2010 in all three schemes, whilst pleuropneumonia has been decreasing. Prevalence of abscesses in the lung has decreased in England, Wales and Northern Ireland but has increased in Scotland. This analysis highlights the value of surveillance schemes based on abattoir pathology monitoring of four respiratory lesions. The outputs at scheme level have significant value as indicators of endemic and emerging disease, and for producers and herd veterinarians in planning and evaluating herd health control programs when comparing individual farm results with national averages.

## Introduction

Surveillance of animal disease provides information essential in the protection of animal health and ultimately public health. Through early detection and informed response, surveillance can help to reduce the impact of animal disease on animal production and welfare. Routine monitoring is generally understood to be the systematic (continuous or repeated) measurement, collection, analysis, and interpretation of animal-health and welfare-data in defined populations, when these activities are not associated with a pre-defined risk-mitigation plan—although extreme changes are likely to lead to action [1].

Originally, meat inspection was introduced at the abattoir in the late 1800s when transmission of zoonotic infectious disease through consumption of animal products was first recognised [2]. However, the sensitivity of official routine meat inspection has since been considered imperfect (lacking sensitivity) for animal disease surveillance. As a consequence, pig health schemes were proposed to provide an additional integrated system to capture information on more detailed post-mortem inspection, aiming to improve sensitivity [3].

Coordinated industry-wide pig abattoir lesion scoring has been implemented in the United Kingdom (UK) with the development of health schemes: Wholesome Pigs Scotland (WPS) commenced in 2003, and the BPEX Pig Health Scheme (BPHS) and health checks in Northern Ireland both commencing in 2005. These schemes report the presence of macroscopic conditions detected in the slaughtered pigs), many of which have been associated with a reduction in performance traits and consequent increases in production costs. The cornerstone of these health schemes’ success has been the frequent feedback of benchmarked results from routine abattoir inspections to the participating producers and their herd veterinarians, helping to increase their awareness of the occurrence of subclinical diseases in their farms [3]. Scheme data is considered useful at producer level as it can be used to assess the presence, severity and response to interventions for different diseases over time, using macroscopic lesions as a proxy for disease [4,5].

Respiratory conditions represent some of the most important diseases affecting pigs [6]. The two predominant conditions identified in pigs at routine abattoir surveillance in UK are lung consolidation likely to be caused by enzootic pneumonia (EP-like lesions) and pleurisy [7]. Both are associated with significant losses for the pig industry worldwide [8,9]. Enzootic pneumonia-like lesions are reported when the gross pathology observed is consistent with the expected lung lesion caused by enzootic pneumonia; i.e. a confluent consolidation affecting the cranioventral regions of the lungs; with appearance ranging from dark red to greyish pink [10]. The main causal infectious agent is *Mycoplasma hyopneumoniae*, *M*. *hyo*, but recent surveys have emphasised that these lesions might have a multifactorial causation [11,12]. Pleurisy (or pleuritis) is a non-pathognomonic finding denoting inflammation of the pleura. Several infectious agents and management factors have been implicated in its pathogenesis: *Actinobacillus pleuropneumoniae*, *Pasteurella multocida*, *Mycoplasma hyopneumoniae*., *Mycoplasma hyorhinis*, swine influenza and *Haemophilus parasuis* [13–15], porcine reproductive and respiratory syndrome virus (PRRSV), porcine circovirus type 2 (PCV2) [12] are reported to be the main infectious agents. Managemental and environmental factors have also been identified as risk factors for pleurisy, such as no all-in-all-out flow, rearing pig with an age difference of more than one month in the same airspace or repeated mixing pigs during the rearing phase [5]. Other respiratory conditions often included in surveillance schemes include pleuropneumonia (acute—PPA and chronic—PPC), viral-like pneumonia lesions and lung abscesses and the same infectious agents are frequently implicated as for pleurisy. The spread of respiratory infections between farms is mainly by the movement of carrier pigs and by fomite transmission [16]. The presence of one or more lung abscesses may be associated with secondary bacterial infection, such as tail biting ascending infections [17].

This study compares the prevalence of four respiratory conditions assessed by the three Pig Health Schemes in UK, assessing the seasonal variations and year trends associated with the conditions in each scheme. The aim is to highlight and draw the attention of respective stakeholders to conditions that may not have responded to control efforts, conditions that may be the target of future control measures and areas where further research is needed. The study also highlights the differences in prevalence for each condition across the countries covered by the schemes—England and Wales (E&W), Scotland and Northern Ireland (NI).

## Materials and Methods

### Data sources

The BPEX Pig Health Scheme (BPHS) (for England and Wales), the Wholesome Pigs Scotland (WPS) (for Scotland), and the Northern Ireland (NI) health and welfare surveillance scheme gather information on several pig health and welfare conditions assessed at abattoirs. Data collected by these schemes between July 2005 and December 2012, from 4,420 slap marks in 46,321 batches of pigs supplied to 28 abattoirs spread across the UK, were used in this investigation. In total 2,061,779 pigs were examined. Slap mark is the herd mark that is tattooed on a pig and identifies farm of origin which is a legally required official reference for each pig farm. In some cases a farm can have more than one slap mark but it was not possible to identify those cases in our analysis so it was assumed that each slap mark will roughly correspond to a farm. Table 1 describes the respiratory conditions assessed and the scoring system used for each one of them.

**Table 1:** Description of the respiratory conditions assessed at abattoir and the scoring system used for each scheme.

Lesion	Pathological lesion	Scoring system		
		BPHS	NI H&W	WPS
Enzootic pneumonia (EP)-like lesions	A red-tan-grey discolouration, and consolidation affecting cranioventral regions of the lungs in a lobular pattern	Represent the approximate percentage of lung with consolidation. Scale from 0 to 55 in five steps (Goodwin et al, 1969).	Represent the approximate percentage of lung with consolidation. Scale from 0 to 55 in five steps (Goodwin et al, 1969).	Represent the approximate percentage of lung with consolidation. Scale from 0 to 55 in five steps (Goodwin et al, 1969).
Pleurisy (PL)	Fibrous or fibrinous adhesions on the lung or between the lung and the chest wall	As WPS until June 2008. From June 2008:0 (absence), 1 for discrete areas of pleurisy (“localised”), 2 when lesions greater than 20% of the lung surface area (“extensive”).	Score from 0 (no lesions) to 5. Score of 1 represents a small (25mm diameter) old lesion, while a score of 5 would involve the whole surface of the lung.	Three categories: 0(absence), 1(“mild”- adhesions involving visceral pleura only), 2 (“severe”- adhesions involving the parietal pleura).
Pleuropneumonia (PP) lesions	Focal areas of lung consolidation with overlying pleurisy usually affecting the middle or caudal lobes	Binary: present (acute or chronic) or absent	Binary: present (acute or chronic) or absent	Binary: present (acute or chronic) or absent
Lung abscess	Localised abscesses within lung	Binary: present or absent	Binary: present or absent	Binary: present or absent

For some conditions, the number of positive cases was sparse so consequently data for all conditions were aggregated monthly for each year and farm. For the purpose of this analysis the conditions which were not recorded as binary (EP-like lesions and pleurisy) were transformed to binary score. They were considered present (if score is higher than zero) or absent (if score is equal to zero). Therefore all the cases with disease or cases with gross evidence of respiratory condition were included in the analysis, independently of their severity.

### Study Sample

#### WPS

The Wholesome Pig Scotland scheme commenced in 2003 in Scotland and monitors the incidence of post-mortem pathologies in slaughtered pigs. Twelve conditions are assessed at the abattoir: enzootic pneumonia (EP)-like lesions, pleurisy, pleuropneumonia (PP) lesions, viral-like pneumonia, lung abscess, pyaemia, liver milk spots, hepatic scarring, papular dermatitis, tail damage, peritonitis and pericarditis. Snout score was also assessed between 2003 and 2005. Data were gathered in this scheme by independent assessors who assess every other pig on the slaughter line up to a maximum of 150 per batch. The detailed operation of the scheme has been described previously [18]. Between July 2005 and December 2012, the scheme examined about 170,233 pigs in seven abattoirs supplied by 347 slap marks in 3,087 batches.

#### NI Health and Welfare checks (NI H&W)

The Northern Ireland Pig Regen Ltd. health and welfare checks were performed in selected abattoirs on average quarterly since 2005. Data were not available for 2007. The scheme assesses eight conditions: enzootic pneumonia (EP)-like lesions, pleurisy, pleuropneumonia (PP) lesions, lung abscess, liver milk spots, papular dermatitis, tail damage and pericarditis. Within the 7.5 years period 189,172 pigs were examined in Northern Ireland. These pigs were slaughtered in four abattoirs, supplied by 498 slap marks in 2718 batches.

#### BPHS

The BPEX Pig Health Scheme (BPHS) started in July 2005 with the aim of monitoring the occurrence of post-mortem gross pathology in clinically healthy pigs in England and Wales. The scheme monitors the same conditions as the WPS except snout score. A sample (up to a maximum of 50 pigs) is selected from each batch by assessing every other pig on the slaughter line. The detailed operation of BPHS has been described previously [18]. Within the seven and half years of the scheme’s operation, a total of 1,702,374 pigs supplied to 17 abattoirs (on average the scheme is implemented in 14 abattoirs but our data includes all the abattoirs used in the scheme between 2005 and 2012) from 3,575 slap marks in 40,516 batches were examined.

### Modelling approach

Two approaches were used to study the temporal patterns of the conditions assessed in the three schemes. The semi-parametric generalised additive model was used to obtain the smooth effects of season and trend for each condition in order to visualise the shape or pattern of these effects and compare overall prevalence estimates among schemes. The generalised linear mixed model was then used to quantify these effects and compare differences between annual trend and seasonal effects across schemes. Season was considered as following: December to February as Winter, March to May as Spring, June to August as Summer and September to November as Autumn.

#### Generalised Additive Model (GAM)—Model 1

The semi-parametric generalized additive model (GAM) was fitted in R using the mgcv package [19]. The model has both linear and nonparametric components. The nonparametric components were fitted as smooth functions where the regression curves were estimated empirically without any pre-specified or imposed structure on the data. The mgcv package uses penalized cubic regression splines. The smoothing parameters were chosen using the Generalized Cross Validation (GCV) criterion [20] such that the smoothing parameter with the lowest score is selected.

We assumed that *p*_*itk*_ is the average probability of detecting a positive condition ](or prevalence rate) in month *i* in year *t* and *scheme k* such that

$$Logit\left(p_{itk}\right)=\;\alpha +\;s\left(season,\;by\;scheme\right)+s\left(time,\;by\;scheme\right)+\;scheme$$ 1

The intercept α is the average log prevalence; *s* is a nonparametric smooth spline function which denotes the flexible functional form of the relationship between each covariate and logit of the prevalence rate of each condition. Penalized cubic regression splines were used for the two model terms. The seasonal patterns were accounted for by the smooth effect of *season* while the smooth effect of *time* represents the long term trend effects, where *time* is continuous and given as$:\;time\;=\;year+\;\frac{DOY}{365}$. DOY is day of the year. The differences in seasonality and trend across schemes were fitted as the interaction between season and scheme and between year and scheme respectively. *Scheme* accounts for differences between the three schemes. We adjusted for overdispersion by fitting a quasibinomial distribution.

#### Generalised Linear Mixed Effect model (GLMEM)—Model 2

Model 1 is limited by the fact that it did not account explicitly for the nested structure of the random effects in the data and may underestimate the true variance of the model parameters. Model 2 takes care of this limitation and also enables the quantification of effects.

The value *p*_*it*_ is the binomial parameter that measures the probability of observing a positive condition in season *i*, and year *t* such that

$$Logit\left(p_{it}\right)=\alpha \;+\;f_{1}\left(season\right)+\;f_{2}\left(time\right)+\;\left(season\text{|}year\right)+\;\left(1\;\text{|}abattoir/slapmark\right)+\left(1\text{|}slapmark\right)$$ 2

This model was fitted at scheme level for each of the conditions. Seasonal fixed effects were represented by the trigonometric function ${\;f}_{1}\left(season\right)\;=\;\beta_{1}sin\left(\frac{2\pi t}{T}\right)+\beta_{2}cos\left(\frac{2\pi t}{T}\right)$ where *T* is the period and *t* is the season of the year. The trend effect is given by the polynomial function *f*_2_ over *time*, where *time* is as defined above. The order of the polynomial function for any given condition may differ between schemes. The determination of the form of these functions was based on the shape of the curves obtained from fitting model 1. In order to effectively compare the magnitude of seasonal effect between schemes, we estimated the seasonal amplitude for each condition as:

$$amp=\;\sqrt{\beta_{1}^{2}+\;\beta_{2}^{2}}$$ 3

The positions of the two extreme seasonal values were obtained using:

$$t=\frac{T}{2\pi }\text{arctan}\left(\frac{\beta_{1}}{\beta_{2}}\right)$$ 4

This is the first extreme if *t*>0 and the second extreme is t + T/2. If *t*<0, the two extremes are found at positions *t + T/2* and *t + T* respectively. If *β*_1_>0, the first extreme is the maximum and the second is the minimum and verse versa [21].

Repeated observations were made on each farm (here represented by slap mark) and abattoir over time, it therefore becomes natural to adjust the model for these grouping effects. Hence, model 2 adjusts for these random effects using the term (1|*abattoir*/*slapmark*) which recognises the fact that farms are nested within abattoirs by adjusting for the differences in groups of farms within and between abattoirs. Slap mark random effect was adjusted to account for correlation of repeated batches from each slap mark. Also, year random effect (*season*|*year*) was used in order to allow for different seasonal slope for each year and to capture the differences in variability of effects between years.

## Results

### Prevalence of lesions by scheme

**Fig 1:**
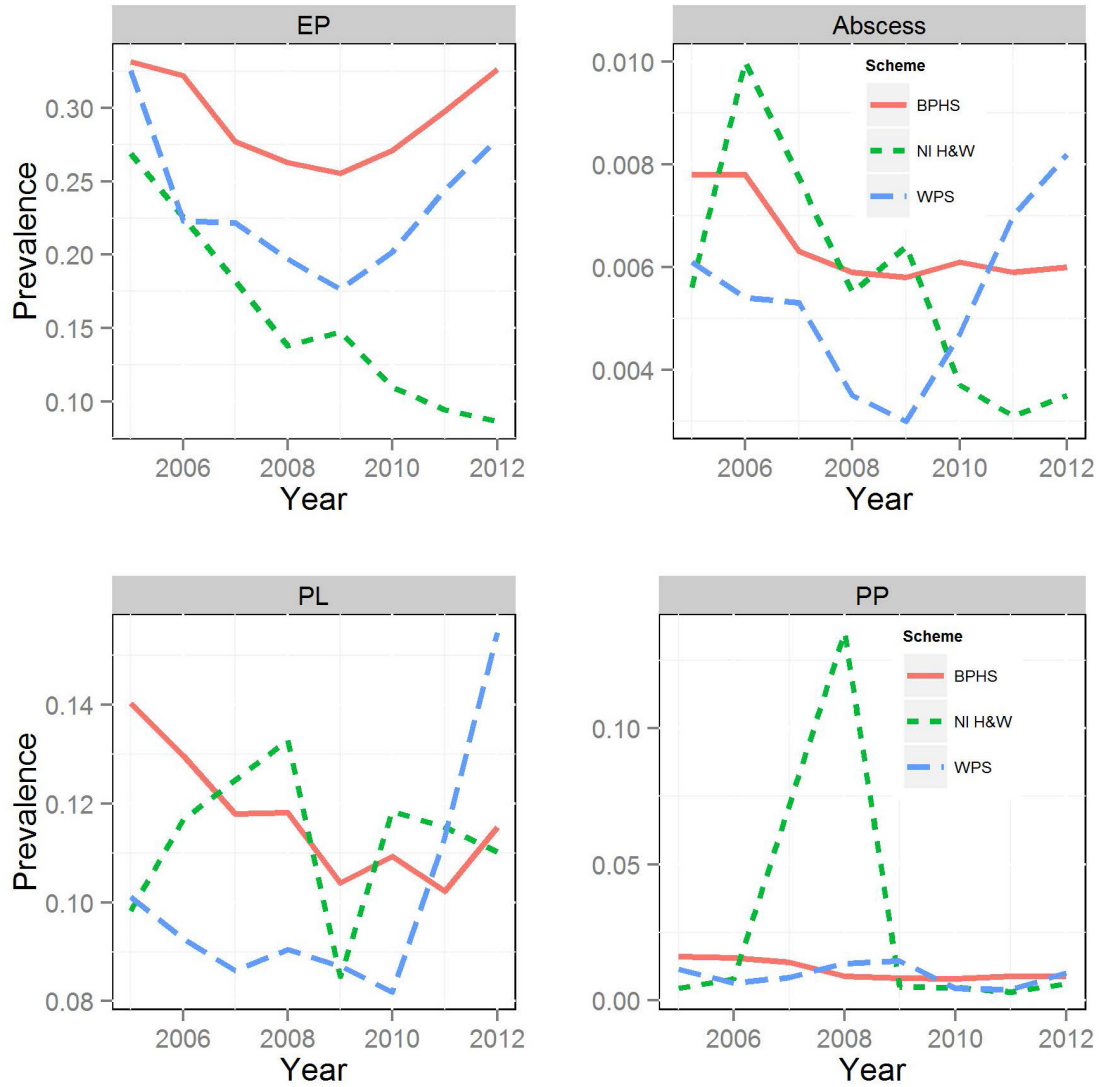
Observed annual average prevalence of respiratory conditions in each scheme. Enzootic pneumonia (EP), pleurisy (PL), pleuropneumonia (PP), and lung abscess (Abscess).

Table 2 describes the proportion of detected positive cases for each condition between July 2005 and December 2012 in the respective scheme. Enzootic pneumonia-like lesions was the most prevalent condition in the three schemes with the highest prevalence observed in England&Wales followed by Scotland (this prevalence was measured at individual level rather than batch level). Average prevalence for the entire study period ranged from 29% in England&Wales, 23% in Scotland to 13% in NI. Pleurisy was the second most prevalent condition with rates ranging on average from 12% in England&Wales, 11% in NI to 10% in Scotland over the study period. The prevalence of pleuropneumonia was relatively low in the three schemes with average prevalence ranging between 1% and 2%. Lung abscess was the least frequently observed condition—approximately 0.5% in each of the three schemes within the period covered by this study. Fig 1 and Table 3, show the yearly prevalence of respiratory lesions across the three schemes.

**Table 2:** Summary of outcomes of the surveillance schemes showing the number of pigs examined and the percentage of cases at animal level for each condition in the three schemes between July 2005 and December 2012.

Scheme	BPHS	NI H&W	WPS	All
Slap marks	3,575	498	347	4,420
Abattoirs	17	4	7	28
Number of batches	40,516	2,718	3,087	46,321
Number of Pigs	1,702,374	189,172	170,233	2,061,779
Enzootic Pneumonia—like lesions (EP-like)	0.290	0.134	0.227	0.271
Pleurisy lesions (PL)	0.115	0.112	0.100	0.114
Pleuropneumonia lesions (PP)	0.011	0.022	0.009	0.011
Lung abscess	0.006	0.005	0.005	0.006

**Table 3:** Annual average prevalence of the four respiratory conditions by scheme.

Schemes	Year	Proportion			
		Enzootic Pneumonia	Pleurisy	Pleuropneumonia	Lung abscess
BPHS	2005	0.3317	0.1403	0.0161	0.0078
	2006	0.322	0.1298	0.0156	0.0078
	2007	0.2772	0.1179	0.0139	0.0063
	2008	0.263	0.1182	0.0089	0.0059
	2009	0.2554	0.1039	0.0081	0.0058
	2010	0.2712	0.1094	0.0079	0.0061
	2011	0.2981	0.1023	0.0088	0.0059
	2012	0.3264	0.1153	0.0089	0.006
NI H&W	2005	0.2691	0.0983	0.0044	0.0056
	2006	0.2255	0.1168	0.008	0.01
	2008	0.1381	0.1328	0.1353	0.0055
	2009	0.1473	0.085	0.0048	0.0064
	2010	0.1099	0.1185	0.0046	0.0037
	2011	0.0943	0.1153	0.0031	0.0031
	2012	0.0866	0.1103	0.006	0.0035
WPS	2005	0.3255	0.1011	0.0115	0.0061
	2006	0.2232	0.0926	0.0063	0.0054
	2007	0.2219	0.0862	0.0084	0.0053
	2008	0.1973	0.0905	0.0134	0.0035
	2009	0.1764	0.0871	0.0144	0.003
	2010	0.202	0.0819	0.0044	0.0047
	2011	0.2444	0.1133	0.0039	0.007
	2012	0.2787	0.1547	0.0100	0.0082

As observed in the preceding paragraph, EP-like lesions were the most common condition recorded and Fig 1 depicts the general trend pattern in each scheme and the average yearly differences among the three schemes. The observed average EP-like lesion prevalence for each year, as shown in Table 3, was highest in England&Wales (range 26%- 33%) followed by Scotland (range 18%– 32%) and NI (range 9%– 27%).

The average annual prevalence of pleurisy was almost the same in England&Wales (range 10%- 14%) and NI (range 9%- 13%) for each year and was higher than that of Scotland until 2011 when prevalence in Scotland was found to exceed that of the other two schemes. Average yearly prevalence in Scotland between 2005 and 2010 was approximately 9% but it increased to 11% in 2011 and to almost 16% in 2012.

For pleuropneumonia the average annual prevalence varied from 0.8% to 1.6% in England&Wales, 0.3% to 13.5% in NI and 0.4% to 1.4% in Scotland. There was a peak of prevalence for pleuropneumonia in NI in 2008.

Lung abscess average annual prevalence varied from 0.6% to 0.8% in England&Wales, 0.3% to 1% in NI and 0.3% to 0.8% in Scotland.

### Analysis of trend and seasonality effects in lesion prevalence by country

**Fig 2:**
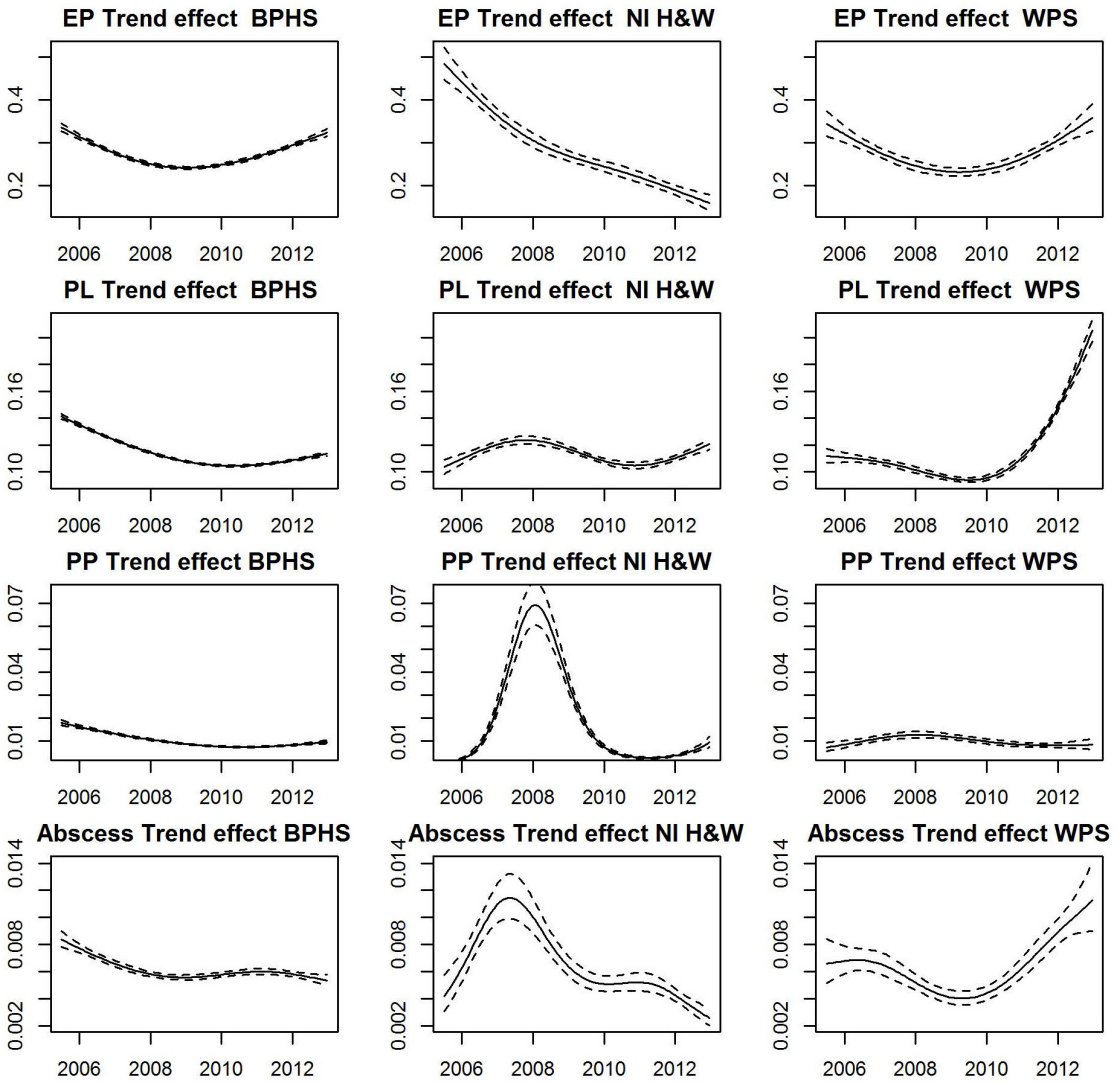
Smooth trend estimates obtained by fitting model 1 to each of the four respiratory conditions. (Enzootic pneumonia-like lesions (EP), pleurisy (PL), pleuropneumonia (PP), lung abscess (Abscess)). The dotted lines are 2 standard deviations (95.4% confidence interval) from the estimated trend (solid line). The y-axis represents estimates of prevalence levels.

**Fig 3:**
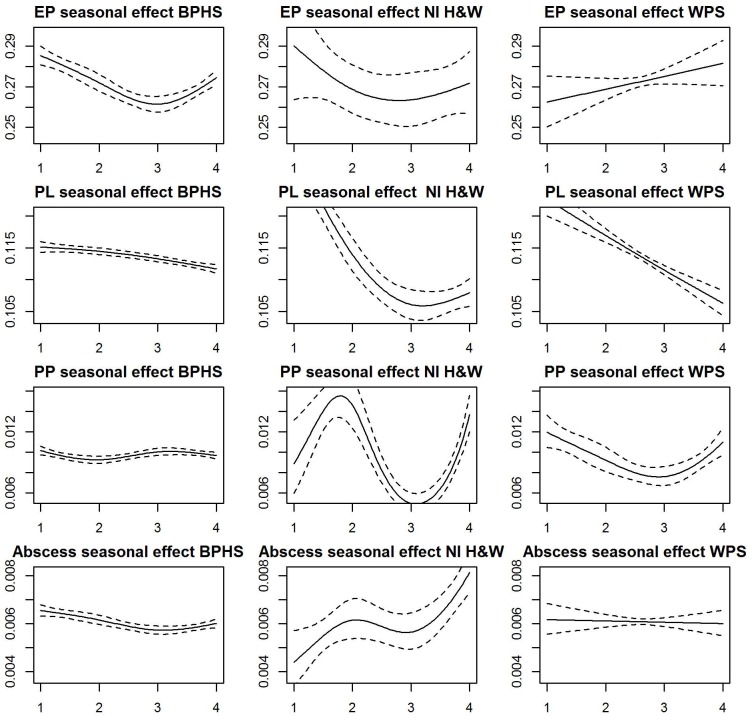
Smooth estimates of seasonal patterns obtained by fitting model 1 to each of the four respiratory conditions. (Enzootic pneumonia-like (EP), pleurisy (PL), pleuropneumoniae (PP), lung abscess (Abscess)). The dotted lines are two standard deviations from the estimated trend (solid line). The y-axis represents estimates of prevalence levels. The x-axis represents the seasons of the year (1 = winter, 2 = spring, 3 = summer and 4 = autumn).

The result obtained by fitting the GAM (model 1) to each condition for the respective scheme and extracting the additive smooth effects of trend and season are displayed in Figs 2 and 3 respectively. Fig 2 depicts the trends of the conditions respectively for the three schemes. Each row in the Figures represents a particular condition and schemes are the columns. The y-axis depicts estimates of levels of prevalence or the probability of observing a positive case for each condition.

#### Trend

The trend in prevalence of EP-like lesions as shown in the first row of Fig 2 was similar for England&Wales and Scotland but differs for NI. While the long term trend of EP-like lesion prevalence in England&Wales and Scotland took a parabolic shape over the study period, the trend declined continuously through the study period in NI. Prevalence fell from 0.48 to 0.1 in NI but trends increased in England&Wales and Scotland after an initial decline between 2005 and 2009. The rate of increase within these recent years was slightly higher in Scotland than England&Wales as seen from the plots.

The pleurisy prevalence trend pattern was similar to that of EP-lesions in England&Wales while in Scotland a marked increase in trend was apparent since 2010. In NI, the trend for pleurisy peaked in 2008 with evidence of a recent smaller peak in 2012.

The pleuropneumonia trend shows a decline since 2008 for Scotland and NI, with evidence of a distinct peak in prevalence in NI in that year. A generally decreasing trend of pleuropneumonia prevalence was observed in England&Wales over the study period.

The trend patterns for lung abscess show a low prevalence across the three schemes. In England&Wales the trend shows a gradual decline. The trend appeared to peak in NI in 2008 before declining while, in Scotland, the trend appeared to be on the increase in the later stages of the study period.

#### Seasonal pattern

The seasonal patterns in prevalence, averaged over the study period, for the respiratory conditions, are represented in Fig 3. The dotted lines are two standard deviations from each point smooth estimate.

Seasonal patterns for prevalence of EP-like lesions were similar in England&Wales and NI where prevalence was highest in winter and lowest in summer. For Scotland, the lowest prevalence was observed in winter and highest in autumn. There is a high degree of uncertainty around the seasonal estimates of EP-like lesions in NI H&W and WPS schemes due to the low frequency and timing of data collection compared to BPHS.

The seasonal shapes of pleurisy prevalence were similar in the three schemes. Prevalence was highest in winter and lowest autumn.

As shown in the third row of Fig 3, average seasonal pattern of pleuropneumonia prevalence was highest during the winter months in Scotland and NI and lowest in the summer. No seasonal pattern was discernable for England&Wales. The prevalence of lung abscess in England&Wales was highest in winter and lowest in summer. Conversely, there was no clear seasonal pattern of lung abscess in Scotland while in NI prevalence was highest in the autumn and lowest in winter.

### Quantifying the effects

This section quantifies the seasonal effects and differences in prevalence between schemes. The last term in model 1 gave estimates of the differences in prevalence of each condition between schemes using BPHS as baseline. Results in Table 4 show the estimates of odds ratios and their corresponding 95% confidence interval relative to BPHS.

**Table 4:** Odds ratio and 95% confidence interval (CI) for the estimate of scheme level differences obtained from parametric terms of the semi-parametric GAM (model 1) for all the conditions between 2005 and 2012 combined.

Conditions	Scheme (95% CI)		
	BPHS	NI health&welfare	WPS
Enzootic pneumonia like (EP)	1.00	0.41(0.39, 0.43)	0.71(0.68, 0.75)
Pleurisy (PL)	1.00	0.99(0.95, 1.03)	0.84(0.81, 0.88)
Pleuropneumonia (PP)	1.00	0.96(0.87, 1.06)	0.84(0.77, 0.91)
Lung abscess	1.00	0.86(0.80, 0.92)	0.82(0.76, 0.88)

A 95% confidence interval that does not include the value 1 was considered statistically significant.

Table 4 indicates that prevalence was significantly higher in England&Wales for most conditions compared to NI and Scotland. The prevalence of EP-like lesions was significantly higher in England&Wales compared to Scotland and NI. The odds of observing EP-like conditions in NI were 0.41 and 0.71 in Scotland compared to England&Wales. Also, more pleurisy lesions were observed in England&Wales than were observed in Scotland and NI. Significantly more cases of lung abscess were observed in England&Wales than was seen in NI and Scotland.

Lower odds of respiratory conditions were observed in NI and Scotland when compared to England&Wales. This was statistically significant for all respiratory conditions in Scotland and for EP-like and lung abscess lesions in NI.

The extent of seasonal effect on within year prevalence for each condition in the respective scheme is examined in this section and shown in Table 5. The effects are represented as odds and the asterisks are indicative of statistical significance at 5% level of significance. The minimum is the time of seasonal troughs while the maximum represents the peaks. These minimum and maximum positions were determined using Eq 4. The contribution of seasonality to overall prevalence for each condition varies across schemes. That is, seasonal effect could be stronger in any given scheme relative to the others for any given condition.

**Table 5:** Seasonal effects obtained by fitting model 2, measured by the size of the amplitude (expressed as odds i.e. exp(*amp*) where *amp* is as defined in Eq 3).

Conditions	Scheme	Seasonal Effect	Minimum	Maximum
Enzootic pneumonia-like (EP)	BPHS	1.05*	Summer	Winter
	NI H&W	1.09	Summer	Winter
	WPS	1.12*	Winter	Autumn
Pleurisy (PL)	BPHS	1.02*	Autumn	Winter/Spring
	NI H&W	1.29*	Autumn	Winter/Spring
	WPS	1.06*	Summer/Autumn	Winter
Pleuropneumonia (PP)	BPHS	1.05	Spring	Summer/Autumn
	NI H&W	1.26*	Summer	Winter
	WPS	1.06	Spring/Summer	Winter
Lung abscess	BPHS	1.09*	Summer	Winter
	NI H&W	1.08	Winter	Autumn
	WPS	1.07	Summer	Winter

The asterisks denote statistical significance at 5% level. The Minimum and Maximum represent seasonal troughs and peaks respectively.

The effect of seasonality on the average annual prevalence of EP-like lesions was strong in England&Wales and Scotland. Average prevalence could change by as much as 5% (i.e. 5% change in the estimate of prevalence between the average prevalence and the maximum or the minimum) in England&Wales and 12% in Scotland due to seasonal effects. Seasonality did not seem to contribute significantly to EP-like lesions prevalence in NI. Seasonal effect on pleurisy prevalence was marginal in England&Wales but was more pronounced in Scotland and NI where seasonality led to about 6% and 29% change in prevalence respectively. Prevalence of lung abscess was estimated to change on average by as much as 9% in England&Wales due to the effect of season. Changes in levels of lung abscess prevalence in Scotland and NI do not seem to be significantly influenced by seasonal effects.

The estimates of seasonal effects in NI should be interpreted with caution given the large level of uncertainty associated with some of the seasonal estimates as shown in Fig 3. The frequency and timing of data collection may have impacted on these estimates.

Repeated samples were taken from batches coming from the same farm (slap mark) within each abattoir in a given year. Model 2 adequately accounted for this hierarchy of variations and the estimates of the random effects are shown in Table 6. Differences in variation as a result of the distribution of slap marks within abattoirs accounted for most of the random variations in prevalence of all the conditions in all the schemes (both accounting for around 60% to 70% of the total random effects). Variation of prevalence rates between abattoirs is not as important as the farm prevalence or the groupings of farm prevalence within abattoirs. Also, except for pleuropneumonia lesions and lung abscess in NI, the year random effects were less influential on the variability of average prevalence compared with the within farms effects and the nested effects of abattoir and farms.

**Table 6:** Summary of Random Effects of year, abattoir and nested farms in abattoirs obtained from model 2.

Conditions	Country	Farm: Abattoir	Abattoir	Farm	year
Enzootic pneumonia-like (EP)	BPHS	0.93 (36)	0.40 (15)	0.80(31)	0.48(18)
	NI H&W	0.73 (20)	0.0 (0)	0.83(22)	2.19 (58)
	WPS	1.25 (38)	0.0 (0)	1.43(43)	0.62 (19)
Pleurisy (PL)	BPHS	0.63 (37)	0.32 (19)	0.66(39)	0.08 (5)
	NI H&W	0.64(31)	0.19 (9)	0.9(44)	0.32 (16)
	WPS	0.59 (30)	0.18 (9)	0.95(49)	0.23 (12)
Pleuropneumonia (PP)	BPHS	1.19 (33)	1.02 (28)	0.97(27)	0.42 (12)
	NI H&W	0.83 (21)	0.00 (0)	1.05(27)	2.04 (52)
	WPS	0.71 (22)	0.76 (24)	1.28(40)	0.43 (14)
Lung abscess	BPHS	0.67(44)	0.35 (23)	0.5(33)	0.13(9)
	NI H&W	1.06 (34)	0.31 (10)	0.85(27)	0.92 (29)
	WPS	0.51 (34)	0.00 (0)	0.72(48)	0.28 (18)

The percentage of contribution to the total variation is shown in brackets.

## Discussion

This study allowed the comparison of slaughter pig pathology collected from similar monitoring programs in Northern Ireland, Scotland and England and Wales. The analysis and comparison of data collected over an eight year period demonstrated the value of such monitoring programmes in identifying trends in the emergence or disappearance of specific lesions, and in identifying seasonal and country-specific effects that might underpin the epidemiology of these lesions. The data taken together form a valuable base of evidence upon which future research hypotheses may be developed and tested.

The annual trend in prevalence of EP-like lesions declined continuously through the study period (2005–2012) in Northern Ireland while, in England&Wales and Scotland, the trend was also initially downward until 2009 but then it reversed in recent years. The reasons for these divergent trends cannot be identified from the current analysis but could be explained by country-specific changes in prevalence of underlying causal agents, changes in pig susceptibility, and or (less likely) changes in environmental factors such as climate, housing, or management features (e.g. during the study period there was an increasing uptake of M. hyopneumoniae protocols to the extent that today nearly all units, except those that are negative for EP, vaccinate piglets and/or weaners against EP in Northern Ireland). Since EP-like lesions are caused by mixed infections, typically including *Mycoplasma spp*. but also including influenza, *P*. *multocida*, and PCV2 [11], exploratory studies would need to consider potential shifts in the prevalence and pathogenicity of all of these organisms. For example, coincident with the start of the increasing trend for EP-like lesions in England&Wales and Scotland, a new strain of influenza (A(H1N1)pdm09) appeared across the UK [22]. However, while the first cases of A(H1N1)pdm09 were detected in Northern Ireland, the downward trend in EP-lesions continued unhindered in that country—although comparative data on the contemporary prevalence of influenza are not available. Country-level changes in pig susceptibility are equally difficult to dissect. A wide range of factors impact on the robustness of pigs’ immune responses to infectious challenge including factors such as genetics or breed, nutrition (both at macro- and micro-nutrient level), prevalence and pathogenicity of immunodysregulatory infections including PRRSV, PCV2, and *M*. *hyopneumoniae*, and finally the proper selection and use of relevant vaccines. Consideration of these factors at country level might permit insights into the observed changes and differences in EP-like lesion prevalence.

Seasonality patterns for EP-like lesions were similar in England&Wales and Northern Ireland where prevalence was highest in the winter months and lowest in the summer, as reported for other European countries where temperature, ventilation, humidity and ultraviolet light levels have been implicated [3,23]. For Scotland, the pattern shifted slightly earlier to peaking in autumn and reaching the nadir in the winter months. However, the large uncertainty associated with the seasonal trend for Northern Ireland and Scotland, due to the low frequency and timing of data collection compared to England&Wales, calls for cautious interpretation.

The trend pattern for pleurisy prevalence in England&Wales and Scotland was similar to that of EP-like lesions in those countries. A general decline was found from 2005 until 2011 when the trend for prevalence started to rise again, especially in Scotland. The reasons behind this apparent higher increase in Scotland should be investigated. The trend pattern of pleurisy in Northern Ireland shows peak in prevalence during 2008. The similarity in trends for prevalence of EP-like lesions and pleurisy in England&Wales and Scotland underlines the close pathophysiological relationship between these lesions and the causal factors for each are likely shared [24,25].

A seasonal effect on pleurisy prevalence was apparent in all three schemes with lower levels of certainty for Scotland and especially Northern Ireland. The lowest levels of prevalence were observed in late summer/autumn for all schemes, while highest prevalences were found in winter/mid spring. This finding is supported by Maes and colleagues [23] who described a similar seasonal pattern. In Scotland the apparent simultaneous trough in late summer in pleurisy prevalence and peak in EP-like lesion prevalence may be explained by the high level of uncertainty. However, the relationship between pleurisy and EP-like lesions may not be simply one based on prevalence; the severity of those EP-like lesions may also be significant and this was not considered in the current study.

The trend in prevalence of PP lesions was similar in Scotland and Northern Ireland where prevalence rose between 2005 and 2008 but declined after 2008. A generally decreasing trend was observed in England&Wales throughout the study period. The seasonality pattern was similar in the three schemes, showing a peak of prevalence in winter months and a lower prevalence in summer months and emphasising the close association between EP-like lesions, pleurisy and pleuropneumonia lesions. The authors do not find any justification for the peak in prevalence in Northern Ireland in 2008 indicating that further research is needed.

The trend patterns for the proportion of cases of lung abscess differed for the three schemes; a decline in the prevalence over the years for England&Wales, an increasing trend in Scotland since 2009, and a decreasing trend in Northern Ireland since 2008 after an initial increase between 2005 and 2008. The reasons behind the increasing trend in Scotland remain obscure to the authors although it is noticeable that the trend is similar for pleurisy in Scotland in recent years. The seasonal pattern of lung abscess is similar as for the other respiratory conditions, except for Scotland (which shows no clear seasonal pattern), with high prevalence in winter months and low prevalence in summer months.

## Conclusion

For Scotland, there was an overall increase of respiratory conditions since 2009. England and Wales have shown the same increasing trend on respiratory conditions since 2009 mainly due to EP-like and pleurisy lesions. While Northern Ireland shows a decrease over time, except for pleurisy. Further research is needed to investigate the reasons for these divergent trends observed between schemes particularly for EP-like lesions and pleurisy. This research would be of valuable use in the future for improving control plans for respiratory disease in each country.

This analysis highlighted the value of surveillance schemes based on abattoir pathology monitoring of respiratory lesions. The outputs at scheme level have significant value as indicators of endemic and emerging disease, and for producers and herd veterinarians in planning and evaluating herd health control programs when comparing individual farm results to the national average.

Similarity of schemes allows for trend comparisons in the prevalence of gross pathological lesions to be made between countries over time, especially between countries with a similar profile in terms of pig production. This enables early detection of increases in prevalence which allows the industry and researchers to investigate the reasons behind them—for example emerging infectious diseases. These schemes are, therefore, valuable assets for endemic disease surveillance, emerging disease early warning, and also welfare outcomes monitoring.
